# In Vitro Investigations into the Potential Drug Interactions of Pseudoginsenoside DQ Mediated by Cytochrome P450 and Human Drug Transporters

**DOI:** 10.3390/molecules29112482

**Published:** 2024-05-24

**Authors:** Zhuo Li, Cuizhu Wang, Jinping Liu, Pingya Li, Hao Feng

**Affiliations:** 1 School of Pharmaceutical Sciences, Jilin University, 126 Xinmin Street, Changchun 130021, China; lizh0205@jlu.edu.cn (Z.L.); wangcuizhu@jlu.edu.cn (C.W.); liujp@jlu.edu.cn (J.L.); lipy@jlu.edu.cn (P.L.); 2 Department of Human Anatomy, College of Basic Medical Sciences, Jilin University, 126 Xinmin Street, Changchun 130021, China

**Keywords:** pseudoginsenoside DQ, cytochrome P450, drug transporters, drug–drug interactions (DDIs)

## Abstract

Pseudoginsenoside DQ (PDQ), an ocotillol-type ginsenoside, is synthesized with protopanaxadiol through oxidative cyclization. PDQ exhibits good anti-arrhythmia activity. However, the inhibitory effect of PDQ on the cytochrome 450 (CYP450) enzymes and major drug transporters is still unclear. Inhibition of CYP450 and drug transporters may affect the efficacy of the drugs being used together with PDQ. These potential drug–drug interactions (DDIs) are essential for the clinical usage of drugs. In this study, we investigated the inhibitory effect of PDQ on seven CYP450 enzymes and seven drug transporters with in vitro models. PDQ has a significant inhibitory effect on CYP2C19 and P-glycoprotein (P-gp) with a half-inhibitory concentration (IC_50_) of 0.698 and 0.41 μM, respectively. The inhibition of CYP3A4 and breast cancer-resistant protein (BCRP) is less potent, with IC_50_ equal to 2.02–6.79 and 1.08 μM, respectively.

## 1. Introduction

**Figure 1:**
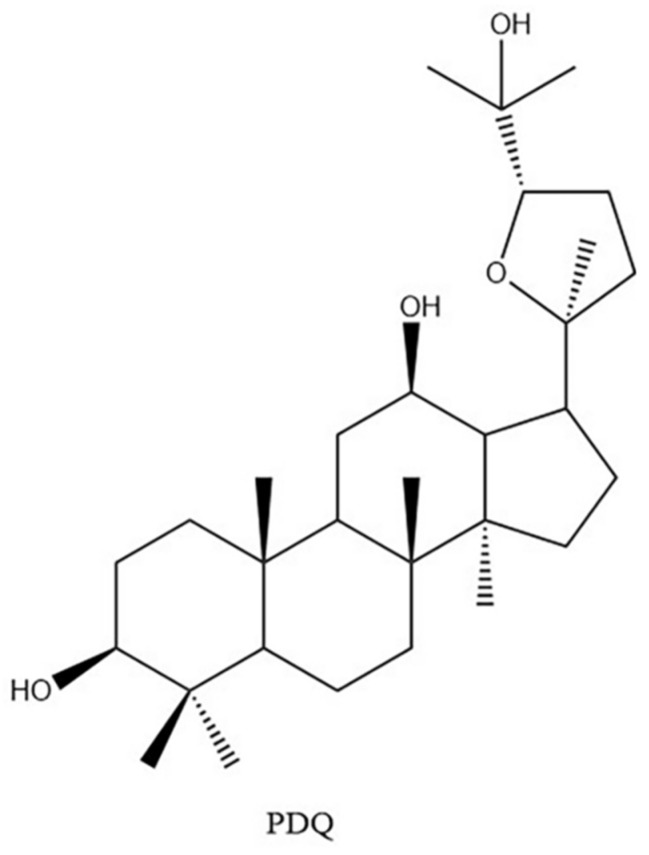
The chemical structure of pseudoginsenoside DQ (PDQ).

Ginsenosides are the main active ingredients of ginseng [1]. The ocotillol-type (20, 24-epoxyside) saponin, which is one type of characteristic ginsenosides of American ginseng, has shown multiple pharmacological activities, such as enhancement of neuronal activity [2], anti-inflammatory effects [3], antiviral activity [4], and anti-tumor activity [5]. Pseudoginsenoside DQ (PDQ), (20S,24S)-dammar-20,24-epoxy-3β, 12β, 25-triol (Figure 1), is one of the semi-synthesized ocotillol-type ginsenosides obtained by oxidative cyclization of protopanaxadiol [6]. PDQ can ameliorate aconitine-induced arrhythmias by shortening the average development time of calcium channels and preventing the effect of potassium channels [7,8]. In the isoproterenol-induced myocardial ischemia rat model, PDQ attenuated injury by suppressing the increase in malondialdehyde content and reducing the activities of glutathione peroxidase and superoxide dismutase [9]. PDQ has not only shown the ability to reverse multi-drug resistance in cancer therapy [10], but also shown activities in preventing the nephrotoxicity of cisplatin by reversing cisplatin-induced changes in signaling pathways [11]. 

To be developed as an anti-arrhythmia drug, the pharmacokinetic properties of PDQ, including absorption, distribution, metabolism, and excretion (ADME) in rats, have been quantitatively studied by our group [12]. The time to peak (T_max_), the concentration at peak (C_max_), and the biological half-time (t_1/2_) of PDQ in rat plasma were 4.0 ± 0.0 h, 3265.12 ± 700.26 ng/mL, and 5.97 ± 0.43 h, respectively [8]. The bioavailability of PDQ in gattate pills was 62.13% [8]. PDQ distributed extensively and quickly to most of the tissues examined after a single oral dose of 30.0 mg/kg in rats, with significantly lower concentrations than in plasma. Additionally, no long-term accumulation of PDQ was observed in tissues [12]. PDQ was primarily excreted through feces, with over 96 h, 55.39% of the cumulative amount in rats [12]. Eleven and five metabolites of PDQ derived from oxidization and dehydration were detected in feces and urine, respectively [12]. The main metabolite pathway was phase I metabolism. However, the potential interactions between PDQ and other drugs mediated by cytochrome P450 (CYP450) enzymes or transporters have not been clarified. 

Both CYP450 enzymes and transporters are key components in modulating drug ADME properties. Evaluating potential drug–drug interactions (DDIs) mediated by CYP450 and transporters is recommended by the U.S. Food and Drug Administration (FDA) to provide information on factors that may influence drug disposition for further clinical study design [13]. The potential interactions of some well-characterized ginsenosides with other drugs have been studied. Ginsenoside Rg3 improved the survival rate and reduced chemotherapy-related adverse events in patients when co-administrated with drugs such as gemcitabine and cisplatin compared to a treatment regime with anticancer drugs alone [14,15]. Ginsenosides Rg1 and Rg3 showed antiplatelet effects against thrombin-induced platelet aggregation in a dose-dependent manner [16]. Compared to aspirin alone, co-administration of ginsenosides increased the plasma concentration of salicylate by twofold in rats [17]. The anticoagulative effect of warfarin reduced when co-administered with ginsenosides, both in rats and in clinical studies [18,19].

The inhibitory effects of some ginsenosides on CYP450 enzymes and transporters have been evaluated to predict potential DDIs. Ginsenosides Rb1, Rc, and Rd strongly inhibited organic anion-transporting polypeptide 1B1 (OATP1B1) and OATP1B3, with IC_50_ values ranging from 0.2 to 4.6 μM, while Rg1 and Re showed weaker inhibition of these transporters, with IC_50_ values ranging from 39.4 to 133 μM in vitro [20]. Ginsenoside Rb1 significantly inhibited CYP2C9, with an IC_50_ value of 2.4 μM [21]. Additionally, ginsenosides may affect metabolizing enzymes and transporters by altering their mRNA expression. Bogacz et al. reported that repeated administration of ginsenosides increased the expression of CYP2D1 and CYP3A2 in rat liver [22]. Regarding drug transporters, the mRNA levels of organic anion transporter (OAT) 1 and OAT3 in the kidney and P-glycoprotein (P-gp) in the liver increased after repeated dosing of ginsenosides in a dose-dependent manner in mice [23]. 

There are numerous DDIs related to cardiovascular medications mediated by CYP450 enzymes and transporters [24]. Understanding the impact of PDQ on metabolizing enzymes and transporters can help minimize potential adverse reactions in clinical use. Therefore, in this study, we evaluated the inhibitory effects of PDQ on seven CYP450 enzymes (CYP1A2, CYP2B6, CYP2C8, CYP2C9, CYP2C19, CYP2D6 and CYP3A4) and seven drug transporters (OAT1, OAT3, organic cation transporter (OCT) 2, OATP1B1, OATP1B3, breast cancer-resistant protein (BCRP), and P-gp) using an in vitro model. We determined the concentrations of PDQ at which the activity of CYP450 enzymes or transporters is inhibited by half (IC_50_), and we also predicted the potential of PDQ to cause CYP450- or transporter-mediated DDIs. These findings will serve as theoretical references for further clinical studies.

## 2. Results

### 2.1. Inhibitory Effects of PDQ on CYP450 Enzymes

#### 2.1.1. Inhibition of Positive Control Inhibitors against CYP450

**Figure 2:**
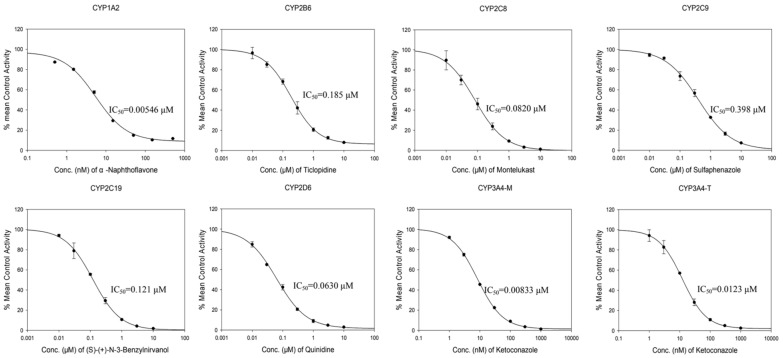
The inhibitory effects of selected positive control inhibitors on CYP1A2, CYP2B6, CYP2C8, CYP2C9, CYP2C19, CYP2D6, CYP3A4 in human liver microsomes (HLM). Data are presented as means ± SD (N = 3).

To evaluate the reliability and reproducibility of the CYP450 inhibition assay, we measured the inhibitory effects of selected positive control inhibitors of each CYP450 enzyme in human liver microsomes (HLM, Table 1, Figure 2). The potency of inhibition was estimated by observing the alteration in concentration of metabolites of CYP450 substrates (Table 1). The IC_50_ values were consistent with previous records from our group, indicating the reliability and consistency of the assay.

Specifically, *α*-naphthoflavone, ticlopidine, montelukast, sulfaphenazole, (S)-(+)-n-3-benzylnirvanol, and quinidine inhibited CYP1A2, CYP2B6, CYP2C8, CYP2C9, CYP2C19, and CYP2D6 with IC_50_ values of 0.00546, 0.185, 0.082, 0.398, 0.121 and 0.063 μM, respectively. For CYP3A4, two substrates, midazolam and testosterone, were employed. The IC_50_ values of the positive control inhibitor of CYP3A4, ketoconazole, were 0.00833 and 0.0123 μM, respectively.

#### 2.1.2. Inhibition Effects of PDQ on CYP450 Enzymes

**Figure 3:**
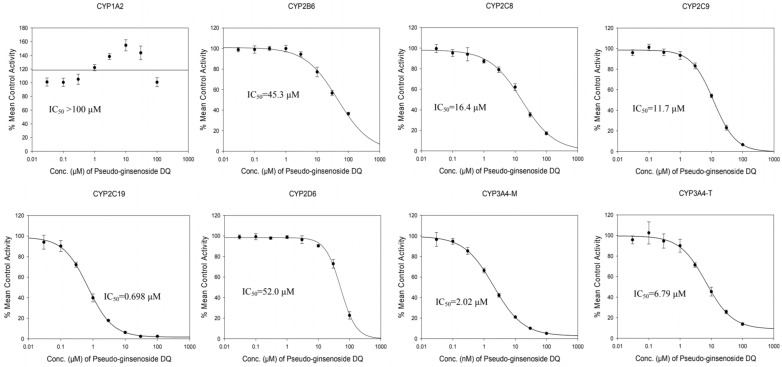
The inhibitory effects of PDQ on CYP1A2, CYP2B6, CYP2C8, CYP2C9, CYP2C19, CYP2D6, and CYP3A4 in HLM. Data are presented as means ± SD (N = 3).

The inhibitory effects of PDQ on the catalytic activities of seven CYP450 enzymes were determined in HLM (Table 1, Figure 3). PDQ exhibited potent inhibition of CYP2C19-catalyzed hydroxylation with an IC_50_ of 0.698 μM. Additionally, PDQ inhibited the hydroxylation of midazolam and testosterone by CYP3A4, with IC_50_ values of 2.02 and 6.79 μM, respectively. 

PDQ demonstrated weak inhibitory effects of CYP2B6, CYP2C8 and CYP2C9, with IC_50_ values of 45.3, 16.4 and 11.7 μM, respectively. However, PDQ did not significantly inhibit the catalytic metabolic activity of CYP2D6 (IC_50_ = 52.0 μM). Interestingly, PDQ activated the de-ethylation of α-naphthoflavone catalyzed by CYP1A2 within the range of 0.03 to 30.0 μM. At the highest tested dose of PDQ, the percentage mean control activity of CYP1A2 was 100.8%, indicating that the IC_50_ was greater than 100 μM.

### 2.2. Inhibitory Effects of PDQ on Drug Transporters

Drug transporters mediate the cellular uptake and efflux of their substrates. Therefore, cell lines overexpressing a drug transporter can be utilized to investigate inhibitors of the transporter by assessing changes in substrate uptake. For each drug transporter, we selected a well-characterized inhibitor as the positive control. Radiolabeled substrates were used to indicate the decrease in transport efficiency when the drug transporters were inhibited by PDQ or the control inhibitors.

The MDCK II-MDR1 cell line is a well-established in vitro model for studying P-gp-mediated efflux. These transporters are polarized, expressed on the apical sides (AP sides) of the monolayer of MDCK II-MDR1 cells. We can investigate the extent to which PDQ affects P-gp-mediated substrate transport from the AP sides to the basolateral sides (BL sides) by assessing the concentration of ^3^H-digoxin in the AP sides compartment.

**Figure 4:**
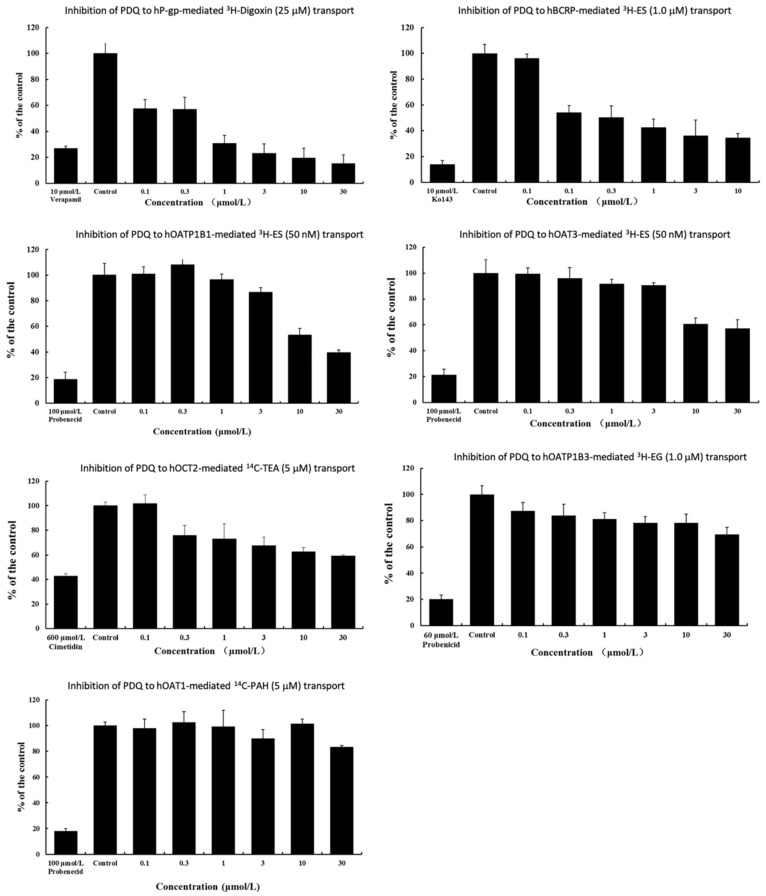
The inhibitory effects of PDQ on seven drug transporters expressed as a percentage inhibition of substrate transport. The negative controls represent 100% transport activities obtained with no PDQ or other inhibitors added. The concentrations of PDQ ranged from 0.1 to 30 μM.

The disintegrations per minute (DPM) value in the AP side of the polarized cells gradually decreased with increasing doses of PDQ (Table S1), indicating inhibition of ^3^H-digoxin transport. PDQ exhibited significantly lower DPM values at 10 μM (427.0) and 30 μM (361.3) compared to the positive control Verapamil at 10 μM (635.3) (Figure 4, Table S1). 

The percentage inhibition of P-gp was 15.3% when PDQ was at 30 μM. The half-inhibitory concentration of PDQ (IC_50_) on P-gp was 0.41 μM, indicating a strong inhibitory effect of PDQ on P-gp (Table 2).

The results of the sf9-BCRP vesicular transport assay showed that the transport of estrone [6,7-^3^H(N)] sulfate-ammonium salt (^3^H-ES), the substrate of BCRP, decreased with increasing PDQ concentration. At the highest tested concentration of PDQ, the percentage inhibition of BCRP was 34.6% (Table S2). PDQ is less potent than the control inhibitor Ko143 (Figure 4, Table S2), while the IC_50_ of PDQ is 1.08 μM (Table 2).

When testing the inhibitory effect against OATP1B1, PDQ showed moderate potency. The percentage inhibition reached 39.6% at the highest tested dose (30 μM) (Figure 4, Table S3). The IC_50_ of PDQ on OATP1B1 is 13.06 μM (Table 2). PDQ shows weak inhibition of OAT3, OCT2, OATP1B3 and OAT1. The percentage inhibition at the highest tested dose were 57.2%, 59.1%, 69.5% and 83.5%, respectively (Figure 4, Tables S4–S7).

## 3. Discussion

The pathogenesis of cardiovascular diseases is complex, and there is a high probability of other complications [25]. Hence, potential drug interactions should be considered if combination treatment with drugs is necessary. CYP450 enzymes are the major metabolic enzymes of many commonly used drugs, such as calcium channel blockers, statins, and proton pump inhibitors [26]. Exogenous substances taken in combination with therapeutic drugs may inhibit CYP450 enzymes, which are a major source of drug–drug interactions. When CYP450 enzyme activities are inhibited, it may induce a decrease in the first-pass metabolism of the therapeutic drug and an increase in blood drug concentration, thereby increasing the risk of drug toxicity, which has a strong correlation with clinical treatment effects [27]. For instance, statins, such as atorvastatin, which are mainly metabolized by CYP3A4, may compete with clopidogrel and reduce the antiplatelet effect of clopidogrel [28].

PDQ is a potent inhibitor of CYP2C19, with an IC_50_ of 0.698 μM. CYP2C19 is responsible for the metabolic activation of the prodrug clopidogrel, which can be metabolically activated into an active 2-oxygen product [29,30]. Clopidogrel reduces cardiovascular morbidity and mortality in patients with CAD, especially those undergoing percutaneous coronary intervention (PCI) [31]. Moreover, CYP2C19 gene mutation can change its metabolic activities and lead to cardiovascular events in patients with CHD. CYP2C19 gene polymorphism has been proved to be a compelling predictor of clopidogrel resistance, which can lead to treatment failure in CHD [32]. Patients with loss-of-function allelic variants (CYP2C19∗2 and CYP2C19∗3) are more prone to thromboembolic events [33]. Due to the strong inhibitory effect of PDQ on CYP2C19, the activation of the prodrug clopidogrel will be affected. As a result, this will reduce the effective concentration of clopidogrel in plasma, tissues or target organs and affect the efficacy of clopidogrel when co-administered with PDQ. Thus, the dosage of CYP2C19-metabolized drugs should be adjusted when combined with PDQ to maintain their efficacy.

It is worth noting that PDQ has a moderate inhibitory effect on CYP3A4 (midazolam, IC_50_ = 2.02 μM and testosterone, IC_50_ = 6.79 μM). CYP3A4 has a large active site and its substrates are usually lipophilic compounds [34,35]. Mirghani et al. confirmed that the anti-arrhythmic drug quinidine is metabolized by CYP3A4 [36]. Nifedipine, a first-generation calcium antagonist, is also a probe substrate of CYP3A4. Bian et al. reported that CYP3A4 plays an important role in the production of oxidized nifedipine by analyzing the mRNA and protein levels of human liver CYP3A4 and the rate of oxidation reaction [37]. Therefore, when PDQ is used together with drugs affected by CYP3A4, potential drug interactions should be taken into account to avoid adverse consequences. The efficacy and toxicity of these drugs should be predicted considering the influence of PDQ. 

The results showed that PDQ, at an effective oral dosage (15 mg/kg), had a high probability of mutual inhibition when co-administered with drugs mainly transported by P-gp, BCRP, OCT2, OAT3 and OATP1B1. Thus, the possibility of DDIs should be considered when combining PDQ with these drugs. For instance, amiodarone has been clinically effective in treating arrhythmia for decades [38]. In small intestinal epithelial cells, amiodarone is primarily effluxed from the cells via P-gp transporter [39]. Since PDQ showed potent inhibition of P-gp, the dosage of amiodarone should be adjusted when administering PDQ together with amiodarone.

On the other hand, PDQ is less likely to interact with drugs transported by OATP1B3 and OAT1. Both P-gp and BCRP are highly expressed in the brush border epithelial cells of the small intestine. If PDQ were a substrate or a substrate-competitive inhibitor of P-gp and BCRP, it could affect the bioavailability of PDQ when administered orally. P-gp and BCRP are also expressed in the liver and kidney, which may increase the elimination of PDQ by the liver and kidney. However, further study is needed to determine whether PDQ is a substrate of P-gp and BCRP.

## 4. Materials and Methods

### 4.1. Inhibition of CYP450 Enzymes

**Chemicals**. PDQ (batch: 20130526, purity = 98.2%) was synthesized by our group. Phenacetin (Cat. No. 77440), amodiaquine (Cat. No. A2799), diclofenac (Cat. No. D6899), dextromethorphan (Cat. No. D9684), testosterone (Cat. No. 86500), acetaminophen (Cat. No. A7085), 4′-hydroxydiclofenac (Cat. No. 32412), 4′-hydroxymephenytoin (Cat. No. H146), dextrorphan (Cat. No. UC205), 6β-hydroxytestosterone (Cat. No. H2898), α-naphthoflavone (Cat. No. N5757), ticlopidine hydrochloride (Cat. No. T6654), sulfaphenazole (Cat. No. S0758), quinidine (Cat. No. Q0750), NADPH (Cat. No. N7505) and DMSO (Cat. No. 34869) were purchased from Sigma Aldrich (St. Louis, MO, USA). Bupropion (Cat. No. B689625), (S)-mephenytoin (Cat. No. M225000), midazolam (Cat. No. M343000), hydroxybupropion (Cat. No. H830675), N-desethylamodiaquine (Cat. No. D288826), 1′-hydroxymidazolam (Cat. No. H948420), montelukast sodium (Cat. No. M568000), (S)-(+)-N-3-benzylnirvanol (Cat. No. B285775), ketoconazole (Cat. No. K186000), acetaminophen-d_4_ (Cat. No. A161222), hydroxybupropion-d_6_ (Cat. No. H830677), N-desethylamodiaquine-d_5_ (Cat. No. D288827), 4′-hydroxydiclofenac-d_4_ (Cat. No. H825227), (±)-4′-hydroxymephenytoin-d_3_ (Cat. No. H944877), dextrorphan-d_3_, tartrate (Cat. No. D299487) and 1′-hydroxymidazolam-^13^C_3_ (Cat. No. H948422) were obtained from TRC (Toronto, ON, Canada). 6β-hydroxytestosterone-d_7_ (Cat. No. 451009) was purchased from Corning (Corning, NY, USA). HPLC grade acetonitrile and methanol were from Merck (Darmstadt, Germany). HPLC grade formic acid (Cat. No. A13285) was obtained from Alfa Aesar (Ward Hill, MA, USA). HPLC grade KH_2_PO_4_ (Cat. No. 10017618) was purchased from Sinopharm Chemical Reagent Co., Ltd. (Shanghai, China). HPLC grade MgCl2 was obtained from Shanghai Shengzhong Fine Chemical (Shanghai, China). 

**Reagents preparation.** The peak concentration (C_max_) of PDQ is 6.5 μM when the effective oral dosage is 15 mg/kg in rats. Thus, the concentration of PDQ was set to 0.03, 0.1, 0.3, 1, 3, 10, 30 and 100 μM to investigate the inhibitory effect of PDQ on the activities of CYP450 enzymes in vitro and to calculate the half maximal inhibitory concentration (IC_50_). The stock solution of PDQ (10 mM) was prepared with methanol and further diluted to the range of 3 µM to 3 mM with methanol to ensure that the solvent of different PDQ samples contained the same amount (1%) of methanol. 

The stock solutions of the positive control inhibitors and the probe substrates of CYP450 enzymes were prepared in methanol. The final concentrations of the positive control inhibitors were listed in Table 3 (1% methanol). The final concentrations of the probe substrates were 75 µM phenacetin, 80 µM bupropion, 2 µM amodiaquine, 10 µM diclofenac, 20 µM (S)-mephenytoin, 10 µM dextromethorphan, 2 µM midazolam and 40 µM testosterone for CYP1A2, CYP2B6, CYP2C8, CYP2C9, CYP2C19, CYP2D6, CYP3A4 and CYP3A4, respectively.

**CYP450 inhibition assay.** The potential inhibitory effects of PDQ on seven CYP450 enzymes (CYP1A2, CYP2B6, CYP2C8, CYP2C9, CYP2C19, CYP2D6 and CYP3A4) were determined in 0.1 mg/mL HLM (Cat. No. 452117, Corning, NY, USA). The assay was conducted in polypropylene 96-well plates (2.2 mL/well, Apricot Designs Inc., Covina, CA, USA). 

The total volume of the reaction is 200 μL. The HLM mixtures (102 μL) containing individual probe substrates (Table 3), KH_2_PO_4_ buffer (100 mM, pH 7.4), and either PDQ or the positive control inhibitors (Table 3) were incubated for 10 min at 37 °C in a shaking incubator. Reactions were initiated by adding 98 μL NADPH (final concentration is 1.3 mM NADPH, 3 mM MgCl_2_ in 100 mM KH_2_PO_4_ buffer). The HLM reaction mixtures were shaken at 37 °C for various times (Table 4). The reaction time of CYP3A4 (midazolam), CYP2C19 and CYP2D6 were 3, 20 and 20 min, respectively, while the reactions of other CYP enzymes were terminated after 10 min. The reactions were quenched by adding 200 μL acetonitrile containing 3% formic acid and specific internal standards (Table 4). After shaking at 1000 rpm for 10 min, the reaction mixtures were centrifuged at 4000 rpm for 20 min. Supernatants were diluted with ultrapure water (Table 4) and shaken for 10 min at 1000 rpm. The samples were stored at 2–8 °C before analysis. The substrates and analytes were analyzed by LC-MS/MS (API4000, Applied Biosystems, Singapore; HPLC, Shimadzu, Japan). The HLM reaction mixtures (containing 1% methanol) without adding inhibitors served as the negative control. Each sample was replicated three times (N = 3). 

**Data analysis.** SigmaPlot (v.11) was utilized for nonlinear regression analysis of the average percentage activity against the concentration of the samples. The IC_50_ was determined by fitting the dependence of the relative remaining activity (y = V_i_/V_0_) to the following three-parameter equation (Equation (1)).

(1) $$y=\frac{V_{i}}{V_{0}}=1-\frac{I^{N}}{{{\mathrm{IC}}_{50}}^{N}+I^{N}}$$

where y is the fractional remaining activity; V_i_ represents the rate in the presence of the inhibitor while V_0_ represents the rate without the inhibitor; N is the so-called hillslope, the parameter determining the steepness of the inhibition curve, and I is the concentration of the inhibitor.

The four-parameter equation (Equation (2)) was employed when the minimum enzyme activity fell within ±10% of V_0_.

(2) $$y=\frac{V_{i}}{V_{0}}=y_{\min}+(y_{\max}-\;y_{\min})\times (1-\frac{I^{N}}{{{\mathrm{IC}}_{50}}^{N}+I^{N}})$$

where y is the fractional remaining activity; V_i_ represents the rate in the presence of the inhibitor while V_0_ represents the rate without the inhibitor; y_max_ represents the maximum enzyme activity; y_min_ denotes the minimum enzyme activity; N is the so-called hillslope, the parameter determining the steepness of the inhibition curve, and I is the concentration of the inhibitor.

If the percentage activities of CYP enzymes exceeded 50% at the highest measured concentration (100 μM), the IC_50_ was labeled as >100 µM.

### 4.2. Inhibition of Human Drug Transporters

**Chemicals.** PDQ (batch: 20150401, purity = 98.2%) was synthesized by our group. Probenecid (Cat. No. P8716), rifampicin (Cat. No. R3501), cimetidine (Cat. No. C4522), verapamil (Cat. No. V4629), estradiol 17-β-D-glucuronide (EG) (BCBB4273V) and estrone sulfate-ammonium salt (ES) (079K4061) were purchased from Sigma (St. Louis, MO, USA) while Ko143 (Cat. No. HY-10010) was obtained from Medchem express (Monmouth Junction, NJ, USA). Estrone [6,7-^3^H(N)] sulfate-ammonium salt (^3^H-ES) (140331), estradiol [6,7-^3^H(N)] 17-β-D-glucuronide (^3^H-EG) (141231) and tetraethylammonium bromide [I-^14^C] (^14^C-TEA) (130311) were purchased from Advance Research Chemicals, Inc (Houston, TX, USA) while para-aminohippuric acid, P-[Glycyl-1-^14^C] (^14^C-PAH) (1558673) was obtained from PerkinElmer (Waltham, MA, USA). The radioactivity was measured with Tri-Carb 2910 TR radioactive liquid scintillator from PerkinElmer (Waltham, MA, USA).

The concentration of PDQ was set to 0, 0.1, 0.3, 1, 3, 10 and 30 μM to investigate the inhibitory effect of PDQ on the seven drug transporters in vitro and to calculate their half-maximal inhibitory concentration (IC_50_). The concentrations of the control drugs and the substrates were stated in the assay details. Working solutions of the control inhibitors, the radiolabeled substrates, and each dose of PDQ were prepared at 200 times (200×) more concentrated solutions with DMSO for further dilution.

**Transporter-overexpressed cell lines.** Human organic anion transporter-overexpressed cell lines MDCKII-OAT1 and S2-OAT3, human organic anion transport polypeptide-overexpressed cell lines HEK293A-OATP1B1 and HEK293A-OATP1B3, human organic cation transporter 2-overexpressed cell line S2-OCT2, and human multi-drug resistance protein MDR1 (or P-gp)-overexpressed cell line MDCKII-MDR1 were gifts from Japan Fuji Biomedical Co., Ltd. (Fuji, Shizuoka Prefecture, Japan). Breast cancer resistance protein-overexpressed vesicles (sf9-BCRP vesicle) were purchased from GenoMembrane Co., Ltd. (Yokohama, Kanagawa, Japan).

**Substrates uptake assay.** In this assay, we studied the inhibition of PDQ against transporters OAT1, OAT3, OCT2, OATP1B1 and OATP1B3 with MDCKII-OAT1, S2-OAT3, S2-OCT2, HEK293A-OATP1B1and HEK293A-OATP1B3 cells, respectively. The radiolabeled substrates for OAT1, OAT3, OATP1B1, OATP1B3 and OCT2 were 5.0 mM ^14^C-PAH, 0.05 mM ^3^H-ES, 0.1 mM ^3^H-ES, 1 mM ^3^H-EG and 5.0 mM ^14^C-TEA, respectively (Table 5). Probenecid (100 mM) was the positive control inhibitor of OAT1 and OAT3, while rifampicin (60 mM) served as the control drug of OATP1B1 and OATP1B3. For OCT2, we used 600 mM cimetidine to compare with the inhibitory effect of PDQ. Two times (2×) more concentrated working solutions of the drugs and the substrate were prepared with Dulbecco’s phosphate-buffered saline (DPBS, Gibco, Grand Island, NY, USA.). Then, an equal volume of 2× drug solution and 2× radiolabeled substrate solution were mixed as the working mix. All reagents were pre-warmed to 37 °C.

The cells were cultured in medium (DMEM with 10% FBS, 1% penicillin and streptomycin) at 37 °C, 5% CO_2_, 95% relative humidity. The cells were digested with 0.05% trypsin/EDTA. The density of the single-cell suspension was adjusted to 2.0 × 10^5^ cells/mL for S2 cells and to 1.5 × 10^5^ cells/mL for MDCK II and HEK293A cells. The cell suspension was then added to the reservoirs of the 24-well plate for culture until full confluency. The medium in the wells was removed, and the wells were washed with DPBS. Then, the plate was incubated for 10 min with pre-warmed DPBS in the wells. After removing DPBS, 500 mL of working mix was added to each well to initiate the substrate uptake process. The reaction time was 5 min for S2-OCT2 cells, while the other four types of cells were incubated with the reaction mix for 2 min. Cold DPBS was used to terminate the substrate transportation. To each well, 400 mL NaOH (0.1 mM) was added to lyse the cells. Then, 100 mL cell lysate was transferred to the counting vials, and 3 mL scintillation cocktail (Aquasol-2, PerkinElmer, Waltham, MA, USA) was added. The radiation in the samples was measured with the Tri-Carb 2910TR scintillation counter (PerkinElmer, Waltham, MA, USA). Each sample was replicated three times (N = 3).

**Vesicular transport assay with the sf9-BCRP model.** Vesicular transport assay was conducted to measure the interaction between PDQ and the efflux transporter BCRP, using sf9-BCRP vesicles (Cat. No, DNAIG03, GenoMembrane, Yokohama, Kanagawa, Japan). The modulation of ^3^H-ES transport reflects the level of the drug-transporter interaction. The concentration of the positive control Ko143 was 10 mM while the concentration of the radiolabeled substrate ^3^H-ES was 1 mM, containing 0.05 mM radioisotope. Ten times (10×) more concentrated stock solutions were prepared for Ko143, ^3^H-ES and every PDQ concentration with the transport buffer (50 mM MOPS-Tris, 70 mM KCl, and 7.5 mM MgCl_2_).

Then, 10× PDQ stocks or 10x Ko143 stock (5 mL) were mixed with 10 mM MgATP (20 mL), 10× substrate stock (5 mL), and 200 mM reduced glutathione (1 mL) to obtain the reaction mix. The MgATP working solution was prepared by diluting the 200 mM stock solution with the transport buffer, while the reduced glutathione solution was made by dissolving glutathione (0.615 g) in 8 mL deionized water and adjusting the pH to 6.8 with 2M NaOH. The drug solution in the reaction mix was replaced with 5 mL of transport buffer as the negative control mix. These reaction mixes were pre-incubated at 37 °C before use. All reagents were thawed on ice, and deionized water was used for the preparation.

The membrane suspension was gently mixed with ice-cold transport buffer and aliquoted to 1.5 mL centrifuge tubes on ice. Each tube contained 10 mL of vesicles (10 mg BCRP) and 9 mL of transport buffer. The vesicle-containing tubes were pre-incubated at 37 °C for 5 min. Then, the reaction mixes were added to the tubes to initiate the assay. To terminate the reaction after 5 min, 400 mL of washing buffer (40 mM MOPS-Tris and 70 mM KCl) was added to each tube on ice. The samples were then transferred to the filter and filtered. The filters were washed with 5 mL of cold washing buffer three times. Subsequently, the filters were placed at the bottom of the counting vials, and the scintillation cocktail was added. The filter and the cocktail were vigorously vortexed to mix. The remaining radiation in the samples was then measured with the scintillation counter. Each sample was replicated three times (N = 3).

**MDCK II-MDR1 drug transport assay**. For the MDCKII-MDR1 drug transport assay, radiolabeled digoxin (^3^H-digoxin) was used as the substrate of P-gp. The MDCK II-MDR1 cells were cultured to confluency in medium (DMEM with 10% FBS, 1% penicillin) at 37 °C, 5% CO_2_ and digested with 0.05% trypsin/EDTA. The density of the single-cell suspension was adjusted to 4 × 10^5^ cells/mL with fresh medium for seeding. To each polycarbonate insert, 0.5 mL cell suspension was added, while to each reservoir of the 12-well plate, 1.5 mL fresh medium was added. The medium was changed every other day for 6–7 days until the formation of a confluent monolayer. Monolayer integrity was checked by measuring transepithelial electrical resistance (TEER) which should be 200~300 Ω·cm^2^.

The positive control was 10 μM verapamil, and the radiolabeled substrate of P-gp was 25 μM ^3^H-digoxin. Two times (2×) more concentrated working solutions of the drugs and the substrate were prepared with Hank’s balanced salt solution (HBSS). Then, equal volumes of 2× drug solution and 2× substrate solution were mixed as the working mix. All reagents were pre-warmed to 37 °C. The medium in the inserts and the reservoir were replaced by HBSS. The plate was incubated for 20 min. After removing HBSS, 1.5 mL of the working mix was added to the basolateral (BL) sides, while 0.5 mL of HBSS was added to the apical (AP) side. The plate was incubated at 37 °C for 15 min while shaking (60 rpm). Then, 100 mL of solution from the AP side was transferred to the counting vials, and 3 mL of scintillation cocktail was added. The radiation in the samples were measured with the scintillation counter. Each sample was replicated three times (N = 3).

**Data analysis**.The percentage inhibition of the transport by drugs was calculated using Equation (3), where U represents the measured radiation of PDQ or positive control inhibitors containing samples, U_c_ represents the negative control in which there are no inhibitors added (U_c_ is considered as 100% transport efficiency), and U_0_ is the measurement of the solvent background.

In = [100 × (U − U_0_)/(U_c_ − U_0_)]% (3)

The results were expressed as mean ± standard deviation (SD) and analyzed with GraphPad Prism 8.0 software (CA, USA). The IC_50_ of PDQ was curve-fitted with the FORECAST function. Statistical significance was calculated using a two-tailed test, and a *p* value < 0.05 was considered as significant.

## 5. Conclusions

The drug interaction potentials of PDQ with CYP450 enzymes and drug transporters were evaluated using in vitro models. PDQ showed potent inhibition of CYP2C19 and P-gp with IC_50_ values of 0.698 and 0.41 μM, respectively. Moderate inhibition of CYP3A4 enzyme and BCRP was also observed. This study provides references for further investigations into PDQ DDIs and contributes important pharmacological data of PDQ.

## Figures and Tables

**Table 1:** The IC_50_ values of selected positive control inhibitors and pseudoginsenoside DQ (PDQ) on the catalytic activities of CYP450 enzymes.

CYP Isoform	Positive Control Inhibitor	Positive Control Inhibitor IC_50_/μM	PDQ IC_50_/µM
CYP1A2	α-naphthoflavone	0.00546	>100.0
CYP2B6	ticlopidine	0.185	45.3
CYP2C8	montelukast	0.082	16.4
CYP2C9	sulfaphenazole	0.398	11.7
CYP2C19	(S)-(+)-N-3-benzylnirvanol	0.121	0.698
CYP2D6	quinidine	0.063	52.0
CYP3A4 (Midazolam)	ketoconazole	0.00833	2.02
CYP3A4 (Testosterone)	ketoconazole	0.0123	6.79

**Table 2:** The table presents the percentage inhibition of selected positive control inhibitors of drug transporters at listed concentrations and the IC_50_ value of PDQ on seven drug transporters. The IC_50_ value was labeled as greater than 30 μM if the percentage inhibition was higher than 50%, indicating that the activity of the drug transporter was not inhibited by half at the highest tested concentration of PDQ.

Transporter	Positive Control Inhibitor (PCI)	PCI Conc./µM	Percentage Inhibition of PCI	PDQ IC_50_/μM
hBCRP	Ko143	10	14.1	1.08
hOAT1	Probenecid	100	18.0	>30
hOAT3	Probenecid	100	21.5	>30
hOATP1B1	Rifampicin	60	18.5	13.06
hOATP1B3	Rifampicin	60	20.2	>30
hOCT2	Cimetidine	600	42.7	>30
hMDR1(P-gp)	Verapamil	10	26.9	0.41

**Table 3:** The table below outlines the probe substrates, positive control inhibitors and metabolic analytes used in CYP450 inhibition assay.

CYP Isoform	Probe Substrate (conc./µM)	Positive Control Inhibitor	Metabolic Analyte
CYP1A2	phenacetin (75)	α-naphthoflavone	acetaminophen
CYP2B6	bupropion (80)	ticlopidine	hydroxybupropion
CYP2C8	amodiaquine (2)	montelukast	*N*-desethylamodiaquine
CYP2C9	diclofenac (10)	sulfaphenazole	4′-hydroxydiclofenac
CYP2C19	*S*-mephenytoin (20)	(*S*)-(+)-N-3-benzylnirvanol	(±)4′-hydroxymephenytoin
CYP2D6	dextromethorphan (10)	quinidine	dextrorphan
CYP3A4	midazolam (2)	ketoconazole	1′-hydroxymidazolam
CYP3A4	testosterone (40)	ketoconazole	*6β*-hydroxytestosterone

**Table 4:** The incubation time of each CYP450 enzyme-metabolizing reaction and the internal standards employed when the reactions were quenched. The supernatants were diluted with water at various volume ratios to obtain the sample for further analyzing by LC-MS/MS.

CYP Isoform	Incubation Time/min	Internal Standard (conc./µM)	Supernatant: Water (*v*/*v*)
CYP1A2	10	acetaminophen-d_4_ (0.6)	1:3
CYP2B6	10	hydroxybupropion-d_6_ (0.32)	1:10
CYP2C8	10	N-desethylamodiaquine-d_5_ (1.8)	1:29
CYP2C9	10	4′-hydroxydiclofenac-d_4_ (0.8)	1:7
CYP2C19	20	(±)4′-hydroxymephenytoin-d_3_ (0.08)	2:1
CYP2D6	20	dextrorphan-d_3_ (0.16)	1:9
CYP3A4	3	1′-hydroxymidazolam-^13^C_3_ (0.4)	1:3
CYP3A4	10	6*β*-hydroxytestosterone-d_7_ (6.4)	1:3

**Table 5:** Drug transporters considered in this study. For each drug transporter, a radiolabeled substrate was used to evaluate the inhibitory effects of PDQ on drug transporters. Positive control inhibitors were also employed for selected drug transporters to provide references for the inhibitory effects of PDQ. The incubation time (reaction time) of the transporter-mediated cellular uptake of radiolabeled substrates is listed.

Transporter	Radiolabeled Substrate	Substrate conc./µM	Positive Control Inhibitor	Inhibitor conc./µM	Incubation Time/min
hBCRP	^3^H-ES	1	Ko143	10	5
hOAT1	^14^C-PAH	5	Probenecid	100	2
hOAT3	^3^H-ES	0.05	Probenecid	100	2
hOATP1B1	^3^H-ES	0.1	Rifampicin	60	2
hOATP1B3	^3^H-EG	1	Rifampicin	60	2
hOCT2	^14^C-TEA	5	Cimetidine	600	5
hMDR1(P-gp)	^3^H-Digoxin	25	Verapamil	10	15

## Data Availability

Data sharing is not applicable to this article as no new data were created or analyzed in this study.

## Supplementary Materials

The following are available online at https://www.mdpi.com/article/10.3390/molecules29112482/s1, Table S1: The inhibitory effects of PDQ at various concentrations and Verapamil (10 μM) on the P-gp activity of transporting ^3^H-Digoxin.; Table S2: The inhibitory effects of PDQ at various concentrations and Ko143 (10 μM) on the BCRP activity of transporting ^3^H-ES.; Table S3:The inhibitory effects of PDQ at various concentrations and Rifampicin (60 μM) on the OATP1B1 activity of transporting ^3^H-ES.; Table S4:The inhibitory effects of PDQ at various concentrations and Probenecid (100 μM) on the OAT3 activity of transporting ^3^H-ES.; Table S5: The inhibitory effects of PDQ at various concentrations and Cimetidine (600 μM) on the OCT2 activity of transporting ^14^C-TEA.; Table S6: The inhibitory effects of PDQ at various concentrations and Rifampicin (60 μM) on the OATP1B3 activity of transporting ^3^H-EG.; Table S7: The inhibitory effects of PDQ at various concentrations and Probenecid (100 μM) on the OAT1 activity of transporting ^14^C-PAH.
