# Supplementary material for: In Vitro Investigations into the Potential Drug Interactions of Pseudoginsenoside DQ Mediated by Cytochrome P450 and Human Drug Transporters
Source: Molecules. 2024 May 24;29(11):2482. doi: 10.3390/molecules29112482 (PMC11173382; doi:10.3390/molecules29112482)
Supplement: Supplementary file 1 [file molecules-29-02482-s001.zip › molecules-2952499-supplementary.pdf]

Supplementary information

In vitro investigations into potential drug interactions of Pseudoginsenoside DQ mediated by cytochrome P450 and human drug transporters

Zhuo Li [1], Cuizhu Wang [1], Jiping Liu [1], Pingya Li [1] and Hao Feng [2,*]

[1] School of Pharmaceutical Sciences, Jilin University, 126 Xinmin Street, Changchun 130021, China; lizh0205@jlu.edu.cn (Z.L.); wangcuizhu@jlu.edu.cn (C. W.); liujp@jlu.edu.cn (J.L.); lipy@jlu.edu.cn (P.L.).
[2] Department of Human Anatomy, College of Basic Medical Sciences, Jilin University, 126 Xinmin Street, Changchun 130021, China; haofeng@jlu.edu.cn.
* Correspondence: haofeng@jlu.edu.cn.

Table S1 The inhibitory effects of PDQ at various concentrations and Verapamil (10 μM) on the P-gp activity of transporting ^{3}H-Digoxin. The negative controls represent 100% transport activities obtained with no PDQ or other inhibitors added.

PDQ concentration / μM	DPM	Transport activity	t-test (p)
0	2359.7±246.3	100.0±10.4%	
0.1	1357.3±167.2	57.5±7.1%	0.0043
0.3	1345.0±217.4	57.0±9.2%	0.0059
1	727.0±150.1	30.8±6.4%	0.0006
3	548.3±100.1	23.2±7.1%	0.0003
10	427.0±28.7	19.5±7.7%	0.0002
30	361.3±91.6	15.3±6.8%	0.0002
Verapamil (10)	635.3±42.0	26.9±1. 8%	0.0003

Table S2 The inhibitory effects of PDQ at various concentrations and Ko143 (10 μM) on the BCRP activity of transporting ^{3}H-ES. The negative controls represent 100% transport activities obtained with no PDQ or other inhibitors added.

PDQ concentration / μM	DPM	Transport activity	t-test (p)
0	5516.7±377.4	100.0±6.8%	
0.1	5303.7±176.6	96.1±3.2%	0.0050
0.3	2995.3±288.8	54.3±5.2%	0.0003
1	2788.3±479.5	50.5±8.7%	0.0006
3	2352.7±355.1	42.7±6.4%	0.0480
10	1997.3±185.1	36.2±3.4%	0.0002
30	1911.0±179.5	34.6±12.1%	0.0369
Ko143（10）	777.7±154.2	14.1±2.8%	0.0003

Table S3 The inhibitory effects of PDQ at various concentrations and Rifampicin (60 μM) on the OATP1B1 activity of transporting ^{3}H-ES. The negative controls represent 100% transport activities obtained with no PDQ or other inhibitors added.

PDQ concentration / μM	DPM	Transport activity	t-test (p)
0	15126.7±1393.1	100.0±9.2%	
0.1	15255.0±875.8	100.9±5.8%	0.8990
0.3	16354.0±875.5	108.1±5.8%	0.2660
1	14584.0±689.5	96.4±4.6%	0.5780
3	13110.3±561.2	86.7±3.7%	0.0810
10	8070.3±754.4	53.4±5.0%	0.0015
30	5983.3±318.4	39.6±2.1%	0.0004
Rifampicin（60）	2798.7±891.8	18.5±5.9%	0.0020

Table S4 The inhibitory effects of PDQ at various concentrations and Probenecid (100 μM) on the OAT3 activity of transporting ^{3}H-ES. The negative controls represent 100% transport activities obtained with no PDQ or other inhibitors added.

PDQ concentration / μM	DPM	Transport activity	t-test (p)
0	7935.7±831.0	100.0±10.5%	
0.1	7893.7±359.2	99.5±4.5%	0.9390
0.3	7623.7±652.1	96.1±8.2%	0.6360
1	7285.3±273.2	91.8±3.4%	0.2670
3	7192.7±157.6	90.6±2.0%	0.2030
10	4919.0±360.3	62.0±4.5%	0.0040
30	4541.3±532.8	57.2±6.7%	0.0040
Probenecid (100)	1709.3±343.6	21.5±4.3%	0.0003

Table S5 The inhibitory effects of PDQ at various concentrations and Cimetidine (600 μM) on the OCT2 activity of transporting ^{14}C-TEA. The negative controls represent 100% transport activities obtained with no PDQ or other inhibitors added.

PDQ concentration / μM	DPM	Transport activity	t-test (p)
0	978.3±94.5	100.0 ± 9.7 %	
0.1	994.3±112.3	101.6±11.5 %	0.8870
0.3	741.3±35.0	75.8±3.6 %	0.0500
1	712.3±115.3	72.8±11.8 %	0.0670
3	661.7±93.5	67.6±9.6 %	0.0330
10	612.0±28.7	62.6±2.9 %	0.0120
30	578.0±29.5	59.1±3.0 %	0.0090
Cimetidine (600)	417.3±44.8	42.7±4.6 %	0.0030

Table S6 The inhibitory effects of PDQ at various concentrations and Rifampicin (60 μM) on the OATP1B3 activity of transporting ^{3}H-EG. The negative controls represent 100% transport activities obtained with no PDQ or other inhibitors added.

PDQ concentration / μM	DPM	Transport activity	t-test (p)
0	3810.3±251.5	100.0±6.6%	
0.1	3332.7±245.2	87.5±6.4%	0.0780
0.3	3200.3±323.1	84.0±8.5%	0.0610
1	3093.3±182.0	81.2±4.8%	0.0160
3	2980.7±186.5	78.2±4.9%	0.0100
10	2985.0±250.2	78.3±6.6%	0.0160
30	2649.0±200.2	69.5±5.3%	0.0033
Rifampicin (60)	769.0±126.5	20.2±3.3 %	0.0005

Table S7 The inhibitory effects of PDQ at various concentrations and Probenecid (100 μM) on the OAT1 activity of transporting ^{14}C-PAH. The negative controls represent 100% transport activities obtained with no PDQ or other inhibitors added.

PDQ concentration / μM	DPM	Transport activity	t-test (p)
0	551.3±16.2	100.0±2.9%	
0.1	540.3±39.2	98.0±7.1%	0.6770
0.3	566.0±44.8	102.7±8.1%	0.6220
1	547.7±68.8	99.3±12.5%	0.9330
3	496.3±38.1	90.0±6.9%	0.0830
10	558.7±19.7	101.3±3.6%	0.6440
30	460.3±5.7	83.5±1.0%	0.0010
Probenecid (100)	99.0±11.6	18.0±2.1%	0.0000
